# Inactivation of tumor suppressor Dlg1 augments transformation of a T-cell  line induced by human T-cell leukemia virus type 1 Tax protein

**DOI:** 10.1186/1742-4690-3-71

**Published:** 2006-10-17

**Authors:** Kojiro Ishioka, Masaya Higuchi, Masahiko Takahashi, Sakiko Yoshida, Masayasu Oie, Yuetsu Tanaka, Sugata Takahashi, Li Xie, Patrick L Green, Masahiro Fujii

**Affiliations:** 1 Division of Virology, Niigata University Graduate School of Medical and Dental Sciences, 1-757 Asahimachi-Dori, Niigata, Japan; 2 Division of Otolaryngology, Niigata University Graduate School of Medical and Dental Sciences, 1-757 Asahimachi-Dori, Niigata, Japan; 3 Division of Pediatrics, Niigata University Graduate School of Medical and Dental Sciences, 1-757 Asahimachi-Dori, Niigata, Japan; 4 Department of Infectious Disease and Immunology, Okinawa-Asia Research Center of Medical Science, Faculty of Medicine, University of the Ryukyus, Okinawa, Japan; 5 Department of Veterinary Biosciences, The Ohio State University, 1925 Coffey Road, Columbus, USA

## Abstract

**Background:**

The interaction of human T-cell leukemia virus type 1 (HTLV-1) Tax1 protein with the tumor suppressor Dlg1 is correlated with cellular transformation.

**Results:**

Here, we show that Dlg1 knockdown by RNA interference increases the ability of Tax1 to transform a mouse T-cell line (CTLL-2), as measured interleukin (IL)-2-independent growth. A Tax1 mutant defective for the Dlg1 interaction showed reduced transformation of CTLL-2 compared to wild type Tax1, but the transformation was minimally affected by Dlg1 reduction. The few Tax1ΔC-transduced CTLL-2 cells that became transformed expressed less Dlg1 than parental cells, suggesting that Dlg1-low cells were selectively transformed by Tax1ΔC. Moreover, all human T-cell lines immortalized by HTLV-1, including the recombinant HTLV-1-containing Tax1ΔC, expressed less Dlg1 than control T-cell lines.

**Conclusion:**

These results suggest that inactivation of Dlg1 augments Tax1-mediated transformation of CTLL-2, and PDZ protein(s) other than Dlg1 are critically involved in the transformation.

## Background

Adult T-cell leukemia (ATL) is an aggressive leukemia that originates mostly from CD4+ T-cells [1-3]. Human T-cell leukemia virus type 1 (HTLV-1) is a causative retrovirus of ATL [4,5]. HTLV-1 immortalizes human CD4+ T-cells *in vitro *and probably does so *in vivo*, but such immortalization is not sufficient for the development of ATL, since only 3–5% of HTLV-1 infection causes ATL after long-latent period of 60–70 years [1-3,6,7]. Multiple genetic and epigenetic changes in HTLV-1-infected cells and deterioration of host immune system during the latent period, are thought to be prerequisite for the development of ATL [3].

HTLV-1 Tax1 is a key player, involved in both T-cell immortalization as well as the leukemogenesis, and it shows transforming activities in various systems [8,9]. Transduction of the *tax1 *gene into peripheral blood mononuclear cells using viral vectors induces interleukin(IL)-2-dependent immortalization of CD4+ T-cells *in vitro *[10,11]. *In vivo*, Tax1-transgenic animals develop various tumors including pre-T-cell leukemia [12-14]. Tax1 also perturbs cellular gene expression, in part, through activation of transcription factors such as NF-*κ*B, serum response factor, and AP-1, thereby inducing the expression of genes encoding cytokines, cytokine receptors, chemokines, and anti-apoptotic factors [8,15-20].

HTLV Type 2 (HTLV-2) is a retrovirus that is similar in many respects to HTLV-1 [21]. For instance, HTLV-2 establishes life-long persistent infection in humans and immortalizes human T-cells in an efficiency equivalent to HTLV-1 *in vitro*. Interestingly, HTLV-2 is not, associated with ATL or related malignancies and has been associated with only a few cases of lymphoproliferative disorders. Recent evidence suggested that the PDZ protein binding motif (PBM) at the C-terminus of Tax1, which is missing in HTLV-2 Tax2, plays a crucial role in the distinct pathogenesis between HTLV-1 and HTLV-2 [8,21-25]. For instance, the transforming ability of Tax1 is much greater than Tax2 in a mouse T-cell line (CTLL-2), and this difference appears to be determined by the PBM [23,25]. A recombinant HTLV-1 with a deletion of the PBM (HTLV-1ΔPBM) failed to establish persistent infection in rabbits, as measured by the lack of antibody responses against HTLV-1 and the absence of HTLV-1 proviruses [24]. Interestingly, HTLV-1ΔPBM can transform human T-cells, although in a less efficient manner than the wild type virus, suggesting that the Tax1 PBM is essential for persistent infection *in viv*o, but dispensable for the transformation of human T-cells.

The PBM of HTLV-1 Tax1 interacts with several PDZ proteins such as Dlg1, the precursor of IL-16, and MAGI-3 [23,26-30]. Among these, Dlg1 is an attractive candidate associated with the transforming activity of Tax1. Dlg originally was isolated from Drosophila and was shown to be a tumor suppressor gene. Loss-of-function mutations in Dlg1 in Drosophila resulted in the neoplastic overgrowth of imaginal disc epithelial cells [31]. Dlg1 also is a tumor suppressor gene in mice, such that Dlg1 heterozygous mice develop B-cell or NK cell lymphomas [32]. Moreover, over-expression of Dlg1 induced cell cycle arrest of a mouse fibroblast cell line NIH3T3, and the arrest was rescued by Tax1 in a PBM-dependent manner [28].

CTLL-2 is a mouse T-cell line, the growth of which is dependent on IL-2. We previously showed that Tax1 abrogates the IL-2-dependent growth phenotype of CTLL-2 [33]. Whereas expression of Tax1 often induces cell growth arrest [34], CTLL-2 is resistant to such Tax1 activity, thereby being a useful tool to examine the transforming activity of Tax1 toward T-cells. In the study reported here, knockdown of D1g1 with RNA interference (RNAi) enhanced the ability of Tax1 to induce IL-2 independence in CTLL-2 cells. Moreover, Dlg1 expression was significantly less in all HTLV-1-transformed T-cell lines compared to HTLV-1-negative cell lines, suggesting that inactivation of Dlg1 is a critical step for transforming activity of Tax1. We will discuss these findings in the context of T-cell transformation by HTLV-1.

## Results

### Dlg1 knockdown augments the ability of Tax1 to induce IL-2-independent growth in CTLL-2 cells

**Figure 1:**
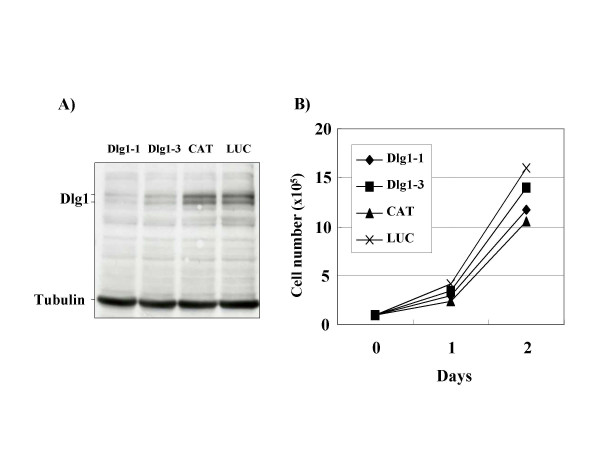
**Dlg1 knockdown in CTLL-2 does not affect the cell growth phenotypes**. (A) CTLL-2 cells infected with lentivirus expressing shRNA for Dlg1-1 (lane 1), Dlg1-3 (lane 2), CAT (lane 3) and LUC (lane 4), were cultured in the presence of blasticidin for more than 10 days. Cell lysates then were prepared and characterized by Western blot analysis using anti-Dlg1 antibody (top) or anti-Tubulin (bottom). (B) The CTLL-2 cells were cultured in the presence of IL-2 and counted by trypan blue exclusion method.

**Figure 2:**
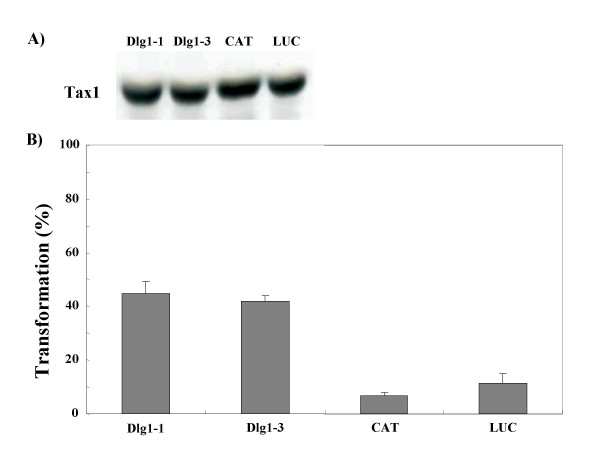
**Dlg1 knockdown augments IL-2-independent cell growth induced by Tax1**. (A) CTLL-2 cells were infected with a lentivirus encoding Tax1. Forty-eight hours after infection, cell lysates were prepared and the amount of Tax1 in the lysates was measured by Western blot analysis using an anti-Tax1 antibody. (B) CTLL-2 cells (Dlg1-1, Dlg1-3, CAT, LUC) infected with Tax1-virus were washed twice with PBS, seeded into 96-well plates at 3 × 10^2 ^cells per well, and cultured in the absence of IL-2 for four weeks. The number of wells containing outgrowing cells was counted under a light microscope. Transformation efficiency (%) was calculated as a ratio of the number of positive wells out of 96 wells. Error bars indicate standard deviations in three independent experiments.

To examine the roles of Dlg1 protein in Tax1-induced IL-2-independent growth of CTLL-2 cells, we established CTLL-2 cells expressing reduced amount of Dlg1 using RNA interference (RNAi). We first constructed lentivirus vectors expressing short hairpin (sh)RNA specific to mouse dlg1 sequences (Dlg1-1, Dlg1-3). Dlg1-1 and Dlg1-3 target distinct sequences of mouse Dlg1 RNA. Two control shRNAs were constructed to target bacterial chloramphenicol acetyltransferase (CAT) and renilla luciferase (LUC) genes, both of which are not expressed normally in mouse T-cells. These viruses were used to infect CTLL-2 cells, and the infected cells were selected by blasticidin for more than 10 days. The established cell lines then were examined for the expression of Dlg1 protein by Western blotting analysis with anti-Dlg1 antibody (Figure 1). Two Dlg1 knockdown cell lines (Dlg1-1, Dlg1-3) expressed a reduced amount of Dlg1 protein relative to two control cell lines (CAT, LUC). These four cell lines grew at equivalent rates in the presence of IL-2 (Figure 1B), and died without IL-2 with similar kinetics (data not shown). Thus, the reduction of Dlg1 expression in CTLL-2 cells did not affect apparent cell growth phenotypes. To examine the effect of Dlg1 knockdown on Tax1-induced IL-2-independent growth, these characterized Dlg1 knockdown cell lines were infected with a lentivirus expressing Tax1 (Tax1-virus) and cultured in the presence of IL-2 for 48 h. Subsequently, the cells were seeded into 96-well plates, followed by further culturing in the absence of IL-2. After more than two weeks, the number of wells with outgrowing CTLL-2 cells was counted using a microscope. All CTLL-2 cells infected with a control lentivirus died within two weeks and did not induce any outgrowing cells (data not shown). Conversely, there was an outgrowth of control CTLL-2 infected with the Tax1-virus (CAT/Tax1, LUC/Tax1) at 7–11% of wells (Figure 2). Similarly, Dlg1 knockdown cells infected with the Tax1-virus (Dlg1-1, Dlg1-3) also had outgrowth with three to six-folds more wells than the controls (CAT/Tax1, LUC/Tax1). The observed differences were not due to reduced Tax1 expression in the control cells, since all four cell lines expressed equivalent amounts of Tax1 protein after the infection as shown by Western analysis (Figure 2A). The augmented Tax1 activity was reproducibly observed in at least nine independent experiments (data not shown). The Dlg1-1 and Dlg1-3 cell lines established by independent experiments also reproduced the high sensitivity to Tax1 transformation relative to control cells (data not shown). Taken together, these results indicate that the reduction of Dlg1 protein in CTLL-2 augmented the ability of Tax1 to induce IL-2 independent growth.

### Dlg1 knockdown doesn't augment Tax1ΔC activity in CTLL-2

**Figure 3:**
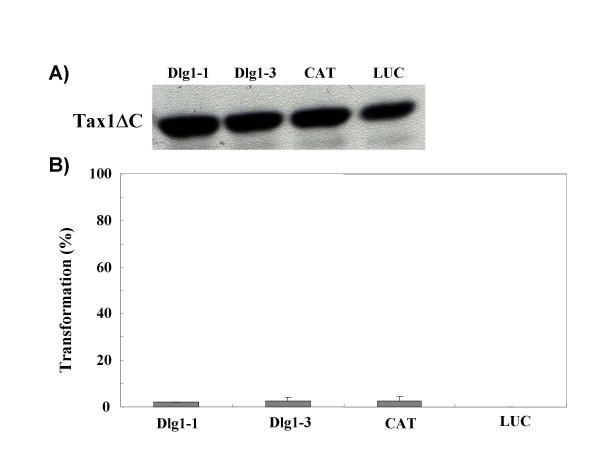
**Dlg1 knockdown does not augment IL-2-independent cell growth induced by Tax1ΔC**. (A) CTLL-2 cells were infected with a lentivirus encoding Tax1ΔC. Forty-eight hours after infection, cell lysates were prepared and the amount of Tax1ΔC in the lysates was measured by Western blot analysis using an anti-Tax1 antibody. (B) CTLL-2 cells (Dlg1-1, Dlg1-3, CAT, LUC) infected with Tax1ΔC-virus were washed twice with PBS, seeded into 96-well plates at 5 × 10^3 ^cells per well and cultured in the absence of IL-2 for four weeks. The number of wells containing outgrowing cells was counted under a light microscope. Transformation efficiency (%) was calculated as a ratio of the number of positive wells out of 96 wells. Error bars indicate standard deviations in three independent experiments.

**Figure 4:**
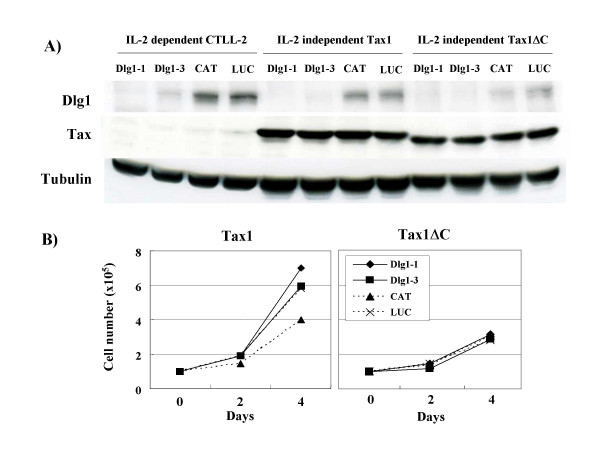
**Low Dlg1 expression in IL-2-independent Tax1ΔC cells**. (A) CTLL-2 cells (Dlg1-1, Dlg1-3, CAT, LUC) were infected with Tax1-virus (lanes 5–8) or Tax1ΔC (lanes 9–12), and cultured in the absence of IL-2 for more than one month in culture flasks. Expression of Dlg1 (top), Tax1 (middle) and Tubulin (bottom) in parental IL-2-dependent CTLL-2 (lanes 1–4), Tax1-transformed CTLL-2 (lanes 5–8) and Tax1ΔC-transformed CTLL-2 (lane 9–12), was measured by Western blot analysis. (B) Cells were cultured in the absence of IL-2 and counted by trypan blue staining. Data are representative of two independent experiments.

We previously showed that Tax1 interacts with Dlg1 through PBM, and the deletion of PBM in Tax1 (Tax1ΔC) greatly reduced IL-2-independent growth mediated by Tax1 in CTLL-2 [25]. These results suggest that wild type Tax1 inhibits the tumor suppressor-like activity of Dlg1 through direct binding via the PBM while Tax1ΔC cannot, resulting in the reduced transforming activity. Therefore, we examined whether Dlg1 knockdown could rescue the transforming activity of Tax1ΔC. Tax1ΔC also induced the outgrowth of control CTLL-2 cells (CAT), but the number of positive wells was much less than that of Tax1, which was consistent with the previous result [25]. It should be noted that 1 × 10^5 ^CTLL-2 cells infected with Tax1ΔC-virus or 3 × 10^2 ^cells with Tax1-virus were seeded per well, indicating that the actual transforming activity of Tax1 versus Tax1ΔC in CTLL-2 cells was much greater than the observed relative difference. Tax1ΔC in the Dlg1 knockdown cells also induced outgrowth, and the number of positive wells was similar to that of the control cells (Figure 3B). These results suggest that inactivation of Dlg1 alone does not explain the difference in transforming activity between Tax1 and Tax1Δ. All four IL-2-independent Tax1ΔC cell lines (Dlg1-1/Tax1ΔC, Dlg1-3/Tax1ΔC, CAT/Tax1ΔC, LUC/Tax1ΔC) grew much more slowly than the IL-2-independent Tax1 cell lines (Figure 4B), suggesting that Tax1 PBM has another function in cell growth as discussed below.

### Reduced expression of Dlg1 in Tax1-transformed cells

**Figure 5:**
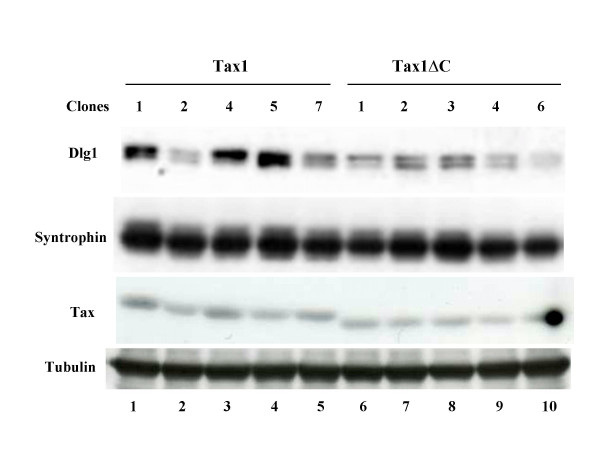
**Low Dlg1 expression in IL-2-independent Tax1ΔC clones**. CTLL-2 cells were infected with Tax1-virus (lanes 1–5) or Tax1ΔC-virus (lanes 6–10), and seeded in 96-well plates in the absence IL-2 for more than one month. The expression of Dlg1 (top), Syntrophin β (second column), Tax1 (third column), or Tubulin (bottom) in Tax1-transformed CTLL-2 clones (lanes 5–8) and Tax1ΔC-transformed clones (lane 9–12), was measured by Western blot analysis using corresponding antibodies.

To confirm the effect of D1g1 knockdown, we examined the expression of Dlg1 in the cells characterized above. Western blot analysis with anti-Dlg1 antibody demonstrated reduced expression of Dlg1 in the Dlg1 knockdown cells (Dlg1-1, Dlg1-3) even after IL-2-independent transformation by Tax1 or Tax1ΔC (Figure 4A). Interestingly, IL-2-independent control cells (CAT, LUC) transformed either by Tax1ΔC or Tax1 expressed less Dlg1 compared to the parental non-transformed cells, and the reduction of Dlg1 was much more prominent in Tax1ΔC cells relative to Tax1. These results support a hypothesis that IL-2-independent transformation of CTLL-2 cells by Tax1ΔC requires reduction of Dlg1 expression. In the above experiments, we used bulk (non-clonal) CTLL-2 cells transformed by Tax1ΔC or Tax1, which were established in culture flasks. To examine this hypothesis further, we evaluated five independent clonal CTLL-2 cell lines transformed either by Tax1ΔC or Tax1 established in 96-well plates and compared the expression level of Dlg1 protein in these cloned cells (Fig 5). All five IL-2-independent Tax1ΔC clones expressed reduced amounts of Dlg1, whereas two out of five Tax1 clones expressed Dlg1 at a level similar to Tax1ΔC. The expression of Syntrophin β, another PDZ protein suggested to interact with Tax1 [27], was expressed equivalently in these cell lines, indicating that reduced expression of Dlg1 in Tax1ΔC is specific. These data lend additional support to the hypothesis that reduced expression of Dlg1 protein is a factor required for Tax1ΔC-induced IL-2-independent transformation of CTLL-2 cells.

**Figure 6:**
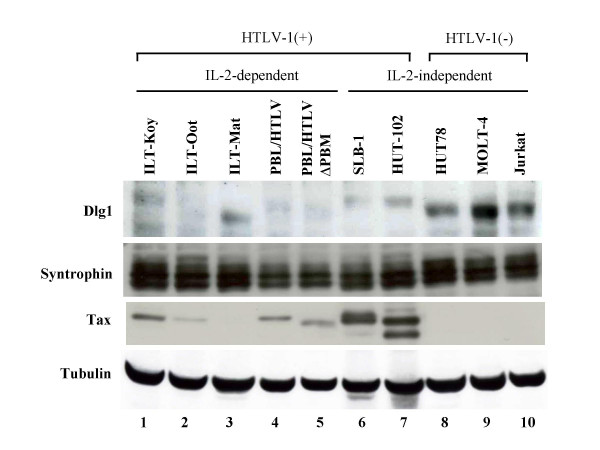
**Dlg1 expression is lower in HTLV-1-transformed human T-cell lines than HTLV-1 negative cell lines**. Cell lysates were prepared from seven HTLV-1 transformed T-cell lines (lanes 1–7) and three HTLV-1 negative T-cell lines (lanes 8–10). The expression of hDlg1 (top), Syntrophin β (second column), Tax1 (third column), or Tubulin (bottom) was measured by Western blot analysis using corresponding antibodies.

We also examined the expression of Dlg1 protein in HTLV-1-transformed T-cell lines (Figure 6). All seven HTLV-1-transformed T-cell lines, including one transformed by HTLV-1ΔPBM with a deletion of the Tax1 PBM, expressed lower amounts of Dlg1 than three HTLV-1 negative human T-cell lines. These results suggest that the Dlg1-low phenotype is preferential for HTLV-1-mediated transformation of human T-cells. The molecular weight of Dlg1 in three HTLV-1-infected T-cell lines (ILT-Koy, SLB-1, HUT-102) that express high amounts of Tax1 was greater than that in HTLV-1 negative T-cell lines, which corresponds to the phosphorylation of Dlg1 in HTLV-1-infected T-cell lines [28]. The biological relevance of phosphorylated Dlg1 in HTLV-1-transformed cells is unclear [25].

### Effect of Dlg1 knockdown on Tax1 transcriptional activity

**Figure 7:**
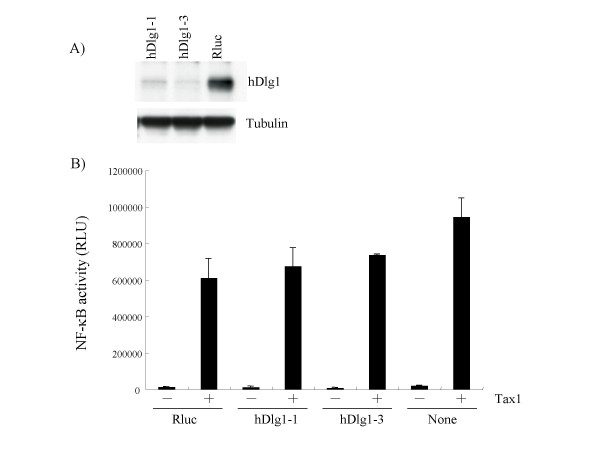
**Dlg1 knockdown little affects transcriptional activity of Tax1**. (A) Cell lysates were prepared from the indicated knockdown cells (hDlg1-1, hDlg1-3, Rluc), and the amounts of hDlg1 protein and Tubulin in cell lysates were measured by Western blot analysis using anti-hDlg1 antibody (top) and anti-Tubulin (bottom), respectively. (B) Jurkat cells (hDlg1-1, hDlg1-3, Rluc) were transfected with *κ *B-Luc plasmid together with pHβPr-1-Tax1-neo plasmid using the lipofection method. Forty-eight hours after transfection, cell lysates were prepared and the luciferase activity in the lysates was measured by a luminometer. Error bars indicate standard deviations.

We next examined the effect of Dlg1 knockdown on Tax1 transcriptional activity. To do so, Jurkat cells were infected with lentivirus expressing shRNA against human dlg1 (hDlg1). Western blotting analysis showed that two hDlg1 knockdown cell lines (hDlg1-1, hDlg1-3) expressed a reduced amount of Dlg1 protein relative to a control cell line (Rluc) targeting a renilla luciferase gene (Figure 7A). These cell lines were then transfected with a Tax1 expression plasmid together with a firefly luciferase reporter plasmid regulated by the NF-*κ*B site, which acts as a Tax1-inducible element, by the lipofection method. Tax1 efficiently activated NF-*κ*B -dependent luciferase activity in two hDlg1 knockdown cells, and the activities were equivalent to those in the control cells (Rluc, None). These results indicates that reduction of hDlg1 protein little affects Tax1 dependent NF-*κ*B activation in T-cells.

## Discussion

HTLV-1 Tax1 interacts with Dlg1 through PBM in various experimental conditions as well as in HTLV-1-infected T-cell lines, and the interaction is well correlated with transforming activity of Tax1 [23,25,26,28,35]. However, it has been unclear whether and how Dlg1 plays a role in Tax1-mediated cellular transformation. Two lines of evidence suggested that inactivation of Dlg1 is a critical step for the transforming activity of Tax1, and Tax1 through PBM inactivates inhibitory activity of Dlg1 to induce transformation of CTLL-2 cells (Figure 2). First, Dlg1 knockdown in CTLL-2 cells increased their ability to be transformed by Tax1 (Figure 2). Second, Tax1ΔC-transformed cells, which were extremely rare to emerge, expressed less Dlg1 than non-transformed cells or Tax1-transformed cells (Figure 4 and 5).

All HTLV-1-transformed T-cell lines expressed low levels of Dlg1 relative to control T-cell lines (Figure 6). However, it is unlikely that reduced Dlg1 expression could be due to Tax1-induced degradation. First, unlike human papilloma virus (HPV) E6, Tax1 expression in the kidney cell line 293T did not induce degradation of Dlg1 [23]. Moreover, a human T-cell line transformed by recombinant HTLV-1ΔPBM containing Tax1ΔC also possessed a low level of Dlg1 protein (Figure 6). Taken together with the findings in CTLL-2 cells, these results suggested that Dlg1 is an inhibitory protein for HTLV-1-induced transformation of human T-cells, and low-Dlg1 expression is preferential for the HTLV-1 Tax1 function.

**Figure 8:**
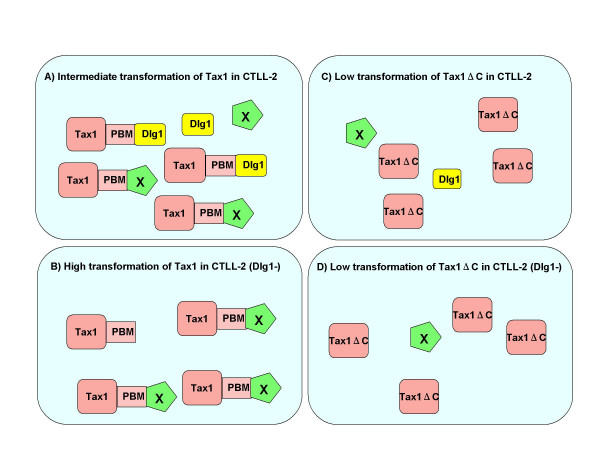
**A model of Tax1 induced transformation**. A) At least two PDZ proteins, Dlg1 and a putative protein X, should be inactivated by Tax1 for the transformation of CTLL2 cells. B) Tax1 inactivates X more efficiently in Dlg1 knockdown cells than in CTLL-2 cells, resulting in the higher transformation rate. C, D) Tax1ΔC selectively transforms CTLL2 expressing low amount of both Dlg1 and X. In this case, cells expressing low amount of X always express low amount of Dlg1.

Dlg1 knockdown in CTLL-2 cells increased the frequency of IL-2-independent transformation induced by Tax1 (Figure 2). Given that Tax1 through the PBM can inactivate Dlg1 function [28], these results indicated that only cells expressing high amounts of Tax1 or reduced amounts of Dlg1 acquire IL-2 independent phenotype (Figure 8A and 8B). This is consistent with the observation that some Tax1-transformed CTLL-2 cells expressed reduced amounts of Dlg1 relative to parental CTLL-2 cells. In addition, this explains why all the human T-cells transformed by HTLV-1 Tax1 with an intact PBM expressed relatively low amounts of Dlg1.

It is unclear how Dlg1 inhibits the transforming activity of Tax1 in CTLL-2 cells, and how such Dlg1 function is inactivated by Tax1. Previous results showed that over-expression of Dlg1 inhibited cell cycle transition from G1 to S phase in the mouse fibroblast cell line NIH3T3, which was overcome by Tax1 in a PBM-dependent manner [28]. On the other hand, Tax1 changes subcellular localization of Dlg1 from detergent soluble fraction to detergent insoluble fraction in HTLV-1-infected T-cell lines and 293T cells, suggesting that Tax1 inactivates Dlg1 function through altering the localization in cells [23]. Together, one possible scenario is that Dlg1 inhibits cell cycle progression of CTLL-2/Tax1, but Tax1 through altering localization of Dlg1 in cells, overcome the cell cycle inhibition to initiate IL-2-independent transformation.

Dlg1 knockdown did not increase transforming activity of Tax1ΔC toward CTLL-2 cells. This finding was initially disappointing to us, since Dlg1 was a major PDZ protein interacting with Tax1 in T-cells (data not shown). This finding, however, suggested that PDZ protein(s) other than Dlg1 inhibits transformation of CTLL-2 by Tax1 (Figure 8). At least two more Tax1-interacting PDZ proteins other than Dlg1 are needed to explain the present data. As discussed above, inactivation of one of the two PDZ proteins should be essential for IL-2-independent transformation of CTLL-2 by Tax1, since Dlg1 knockdown did not enhance the frequency of cells transformed by Tax1ΔC (Figure 3). The other PDZ protein likely influences the rate of proliferation of IL-2-independent Tax1 cells, since IL-2-independent Tax1ΔC cells grew more slowly than IL-2-independent Tax1 cells (Fig 4). However, it should be noted that transformed Tax1ΔC cells exhibited more cell death than transformed Tax1 cells (data not shown). Thus, the latter PDZ protein might regulate apoptosis of T-cells expressing Tax1. There are several Tax1-interacting PDZ proteins, such as MAGI-3 and the precursor of IL-16 [30]. In addition, there are three Dlg1 family members, such as Chapsyn-110 (PSD-93), NE-Dlg (SAP102), and PSD-95 (SAP90) [36], although it is unclear whether they are expressed in T-cells. Therefore, the identification of PDZ proteins other than Dlg1 that are involved in Tax1 function is crucial to elucidate the mechanism of T-cell transformation by HTLV-1.

## Conclusion

The Tax1 PBM is conserved in all known HTLV-1 isolates but not in HTLV-2 isolates. Similarly, the E6 oncoprotein derived from high-risk HPVs, but not low-risk HPVs, has a PBM and interacts with Dlg1. These results strongly suggest that the PBM and the interacting protein(s) play crucial roles in oncogenesis by these viruses. Approximately 12% of Dlg1 heterozygous mice developed B-cell or NK lymphomas, which suggests that Dlg1 is involved in lymphomagenesis, even when its expression is half of that of wild-type mice [32]. Thus, Dlg1 is an attractive candidate regulating not only human T-cell transformation but also ATL leukemogenesis.

## Materials and methods

### Cells and cell growth assay

CTLL-2 is a mouse cytotoxic T-cell line that grows in an IL-2-dependent manner [24,33]. The human T-cell lines used in the present experiments have been characterized previously [23]. ILT-Koy, ILT-Oot, ILT-Mat, PBL/HTLV-1, PBL/HTLV-1ΔPBM are IL-2-dependent HTLV-1-transformed human T-cell lines, while SLB-1 and HUT-102 are IL-2-independent. PBL/HTLV-1 and PBL/HTLV-1ΔPBM were established by recombinant wild type HTLV-1 and HTLV-1ΔPBM with a deletion of PBM in Tax1, respectively [24]. HUT78, MOLT-4 and Jurkat are HTLV-1-negative human T-cell lines. 293T is a human embryonic kidney cell line. SLB-1, HUT-102, HUT78, MOLT-4 and Jurkat were cultured in RPMI1640 supplemented with 10% fetal bovine serum (FBS), 4 mM glutamine, penicillin (50 U/ml), and streptomycin (50 μg/ml) (RPMI/10%FBS). CTLL-2 cells were cultured in RPMI/10% FBS containing 2-mercaptoethanol and 1 nM recombinant human IL-2. IL-2-independent CTLL-2 cells stably expressing Tax1 were cultured in RPMI/10%FBS and 2-mercaptoethanol without IL-2. IL-2-dependent human T-cell lines were cultured in RPMI/20%FBS with 1 nM IL-2. 293T cells were cultured in Dulbecco's modified Eagle's medium supplemented with 10% FBS, penicillin (50 U/ml), and streptomycin (50 μg/ml).

For the cell growth assay, CTLL-2 (10^5^/ml of RPMI/10%FBS) were cultured with or without IL-2 in a 24 well plate. The number of viable cells was counted by the trypan blue exclusion method under a microscope.

### Plasmids and oligonucleotides

pHβPr-1-Tax1-neo is a Tax1 expression vector, which has a β-actin promoter and a neomycin resistance gene as a selection marker. *κ*B-Luc is a luciferase expression plasmid regulated by the *κ*B element of the IL-2 receptor *κ*-chain gene and the minimal HTLV-1 promoter. The lentiviral expression vectors, pSIN-eGFP and CS-CDF-CG-PRE, were kindly provided by Dr. C. Boshoff (Wolfson Institute for Biomedical Research) and Dr. H. Miyoshi (RIKEN Tsukuba Institute), respectively [37]. The lentiviral expression vector pSIN-*bsr*EGFP was constructed by replacing the eGFP gene (a *BamHI – NotI *fragment) of pSIN-eGFP with the *bsr*EGFP gene (an *EcoRI – NotI *fragment) from pkB-*bsr*GFP [38]. eGFP and *bsr*EGFP genes are an enhanced green fluorescent protein gene and a chimeric gene of eGFP with blasticidin S deaminase, respectively. The lentiviral expression vector CS-CDF-CP-PRE was constructed by replacing the eGFP gene (a *NheI – XhoI *fragment) of CS-CDF-CG-PRE with a PCR amplified puromycin-N-acetyl-transferase gene from pIRESpuro3 (Clontech). Dlg1-1, Dlg1-3, hDlg-1, hDlg1-3, CAT, LUC, and Rluc are oligonucleotides used for the construction of short hairpin (sh)RNA-expressing plasmids against mouse dlg1 sequences (nt1092-1111 and nt2391-2410), human dlg1 (hDlg1) sequences (nt2135-2153 and nt2563-2581), chloramphenicol acetyltransferase, and renilla luciferase genes, respectively. The sequences of these oligonucleotides are 5'-ggatggcgagctttaggttggGTGTGCTGTCCccaatctgaagcttgccatccTTTTT-3' for Dlg1-1, 5'-ggatgtttaggagtataagttGTGTGCTGTCCaacttatgctcctgaatatccTTTTT-3' for Dlg1-3, 5'-gaaagaacgagcccgattaTTCAAGAGAtaatcgggctcgttctttcTTTTT-3' for hDlg1-1, 5'-gtgttcagtctgtacgagaTTCAAGAGAtctcgtacagactgaacacTTTTT-3' for hDlg1-3, 5'-gagtggatgccacgacggtttGTGTGCTGTCCaaatcgtcgtggtattcactcTTTTT-3' for CAT, 5'-ggcctttcactgctcctgcgaGTGTGCTGTCCtcgtaggagtagtgaaaggccTTTTT-3' for LUC, and 5'-gcctttcactactcctacgTTCAAGAGAcgtaggagtagtgaaaggcTTTTT-3' for Rluc. The oligonucleotides Dlg1-1, Dlg1-3, CAT, and LUC were cloned into pGEM-U6L, which has a U6 gene promoter under the control of RNA polymerase III. hDlg1-1, hDlg1-3, and Rluc were cloned into pSUPER, a gift from Dr. R. Agami (The Netherlands Cancer Institute), which has a H1-RNA gene promoter. The *EcoRI *fragments containing respective U6 promoter/shRNA or H1 promoter/shRNA sequences then were subcloned into the *EcoRI *site of pSIN-bsrEGFP or CS-CDF-CP-PRE, respectively.

To construct lentiviral expression plasmids for Tax1 (pSIN-EF-Tax), a DNA fragment containing the EF1α gene promoter was amplified from pEFneo [39]. The amplified fragment was exchanged with the SFFV promoter fragment in pSIN-eGFP using *EcoRI *and *BamHI *sites (pSIN-EF-eGFP). To utilize the Gateway recombination system (Invitrogen), the Gateway Reading Frame Cassette A fragment was inserted in the *BamHI *and *NotI *sites of pSIN-EF-eGFP in place of eGFP (pSIN-EF-RfA). The Tax1 and Tax1ΔC coding sequences were subcloned into pENTR/D-TOPO (Invitrogen), and transferred to pSIN-EF-RfA by a Gateway recombination reaction according to the manufacturers' instructions. The Tax1 and Tax1ΔC genes were described previously [23].

### Establishment of knockdown cells

Lentiviruses expressing shRNAs described above were produced according to a three plasmid one shot expression system in 293T cells [40]. These lentiviruses then were used to infect CTLL-2 or Jurkat cells (4 × 10^5^) in a final volume of 2.0 ml RPMI/10%FBS containing 8 μg/ml of polybrene (Sigma) and 1 nM IL-2 for CTLL-2. The infected CTLL-2 and Jurkat cells were cultured in the selection medium containing 14 μg/ml of blasticidin (Invitrogen) or 0.2 μg/ml of puromycin (Sigma) for more than 10 days, respectively. The expression of Dlg1 in the selected cells was examined by western blotting analysis.

### IL-2-independent transformation assay

CTLL-2 cells (4 × 10^5^) were infected with lentiviruses encoding Tax1 or Tax1ΔC in a final volume of 2.0 ml RPMI/10%FBS containing 8 μg/ml polybrene (Sigma) and 1 nM IL-2. At 48 hours after infection, the cells were washed twice with phosphate-buffered saline (PBS), and cultured in RPMI/10%FBS without IL-2. For the 96-well plate assay, the infected CTLL-2 cells were cultured (300 cells/well/0.1 ml for Tax1 or 5000 cells/well/0.1 ml for Tax1ΔC) without IL-2. During the culture period, the medium was changed every three days. After four weeks, the number of wells containing outgrowing cells was counted under a light microscope. Transformation efficiency (%) was calculated as a ratio of the number of positive wells out of 96 wells.

### Western blotting

CTLL-2 cells were lysed with sodium dodecyl sulfate (SDS)-sample buffer consisting of 62.5 mM Tris-HCl (pH 6.8), 2% SDS, 10% glycerol. Protein concentrations of the cell lysates were measured using the DC protein assay kit (Bio-Rad Laboratories). The cell lysates then were treated with 50 mM DTT, 0.01% bromophenol blue and heated at 95^0^C for 5 min. The resultant lysates were subjected to SDS-PAGE containing 8% acrylamide gel for Dlg1 or 10% acrylamide for Tax1, Syntrophin β or Tubulin, and the proteins in the gel were transferred to a nitrocellulose membrane. The membrane was incubated with 5% skim milk for 1 h at room temperature followed by incubation with specific antibodies shown below. After washing, the membrane was treated with a secondary antibody conjugated with horseradish peroxidase. Specific protein bands were visualized using the ECL Western blotting detection system (Amersham Pharmacia Biotech). Antibodies used were anti-human Dlg1 (BD Biosciences), anti-Tax1 (TAXY-7) [41], anti-Syntrophin β (Affinity Bioreagents), and anti-Tubulin (Oncogene).

### Transient transfection and luciferase assays

Jurkat cells in RPMI/10%FBS were seeded at 4 × 10^5 ^cells/well in a 12-well plate. The cells then were cotransfected with the Tax expression plasmid together with *κ*B-Luc by using Transfectin (Bio-Rad Laboratories) according to the manufacturer's instructions. At 48 hours after transfection, cell lysates were prepared from the transfected cells, and the luciferase activity was determined using Luciferase Assay System (Promega) and a luminometer (LUMAT LB9507, Berthold).

## Competing interests

The author(s) declare that they have no competing interests.

## Authors' contributions

KI, MH and MT, LX, SY, and YT carried out the establishing the cell lines and the functional analysis of the cell lines. MO, ST, PG and MF participated in the experimental design, data interpretation, and writing of the manuscript.
